# Postoperative Kinesiophobia in Patients with Acute Type A Aortic Dissection: A Cross-Sectional Study

**DOI:** 10.31083/j.rcm2508304

**Published:** 2024-08-22

**Authors:** Yaqiong Chen, Yanchun Peng, Xizhen Huang, Liangwan Chen, Yanjuan Lin

**Affiliations:** ^1^Department of Nursing, Union Hospital, Fujian Medical University, 350122 Fuzhou, Fujian, China; ^2^Department of Cardiovascular Surgery, Union Hospital, Fujian Medical University, 350122 Fuzhou, Fujian, China; ^3^Key Laboratory of Cardio-Thoracic Surgery (Fujian Medical University), Fujian Province University, 350122 Fuzhou, Fujian, China

**Keywords:** acute type A aortic dissection, movement, kinesiophobia, influencing factor

## Abstract

**Background:**

This cross-sectional study explores postoperative kinesiophobia in patients with acute type A aortic dissection (AAAD), an understudied area. The occurrence of postoperative kinesiophobia and its relation to various factors were investigated.

**Methods:**

Patients diagnosed with AAAD and undergoing surgical treatment from January 2019 to December 2021 were selected through continuous sampling. Kinesiophobia levels were assessed using the Tampa Scale for Kinesiophobia Heart (TSK-SV-HEART). Univariate and multivariate regression analyses were employed to determine factors influencing kinesiophobia.

**Results:**

Out of 264 included patients, the mean postoperative kinesiophobia score was 38.15 (6.66), with a prevalence of 46.2%. Multivariate logistic regression revealed that education level, general self-efficacy, family care index, and facing style reduced kinesiophobia, while avoidance style and yielding style increased it.

**Conclusions:**

Postoperative kinesiophobia prevalence in AAAD patients is high and associated with diverse factors. Medical staff should remain vigilant to potential kinesiophobia during postoperative rehabilitation.

## 1. Introduction

Acute type A aortic dissection (AAAD) is a dangerous condition that usually presents with sudden and severe chest, back and abdominal pain wherein the typical initial symptoms are such that patients feel tearing or impending doom, and undergo shock in severe cases; the detection rate and number of cases have been increasing annually [1]. Advances in medical technology have improved the survival rate of patients with AAAD, and the 1-year survival rate was 94%, and the 2-year survival rate was 92.2% [2]. A database from the International Registry of Acute Aortic dissection (IRAD) [3] revealed that 54.9% of patients with type A dissection lacked physical activity after surgery, and the number showed a gradually increasing trend. Patients with AAAD are still at high risk of postoperative aortic lesions and related cardiovascular events, and their quality of life is lower than that of the normal population [4]. Studies have shown that over 50% of patients with AAAD experience new depression due to lack of physical activity, and more than one-third of patients have reported disability at work [4]. Studies have demonstrated that exercise is a key factor in cardiac rehabilitation (CR), and CR reduces cardiac mortality and risk of hospitalization [5], and improves oxygen uptake and mental health [6, 7]. A study on kinesiophobia in hospitalized patients with acute cardiovascular events also reported that 83% of patients had kinesiophobia [8], which reduced the CR attendance rate [9]. The European Society of Cardiology emphasizes that exercise at a safe level is feasible for patients with heart disease [10]. However, Pasadyn *et al*. [11] investigated 132 postoperative patients with AAAD and found that 36% did not participate in CR treatment, exhibiting low participation and compliance. Studies have shown that high levels of kinesiophobia are associated with a decline in health-related quality of life, muscle strength and physical activity levels [12].

**Fig. 1.**
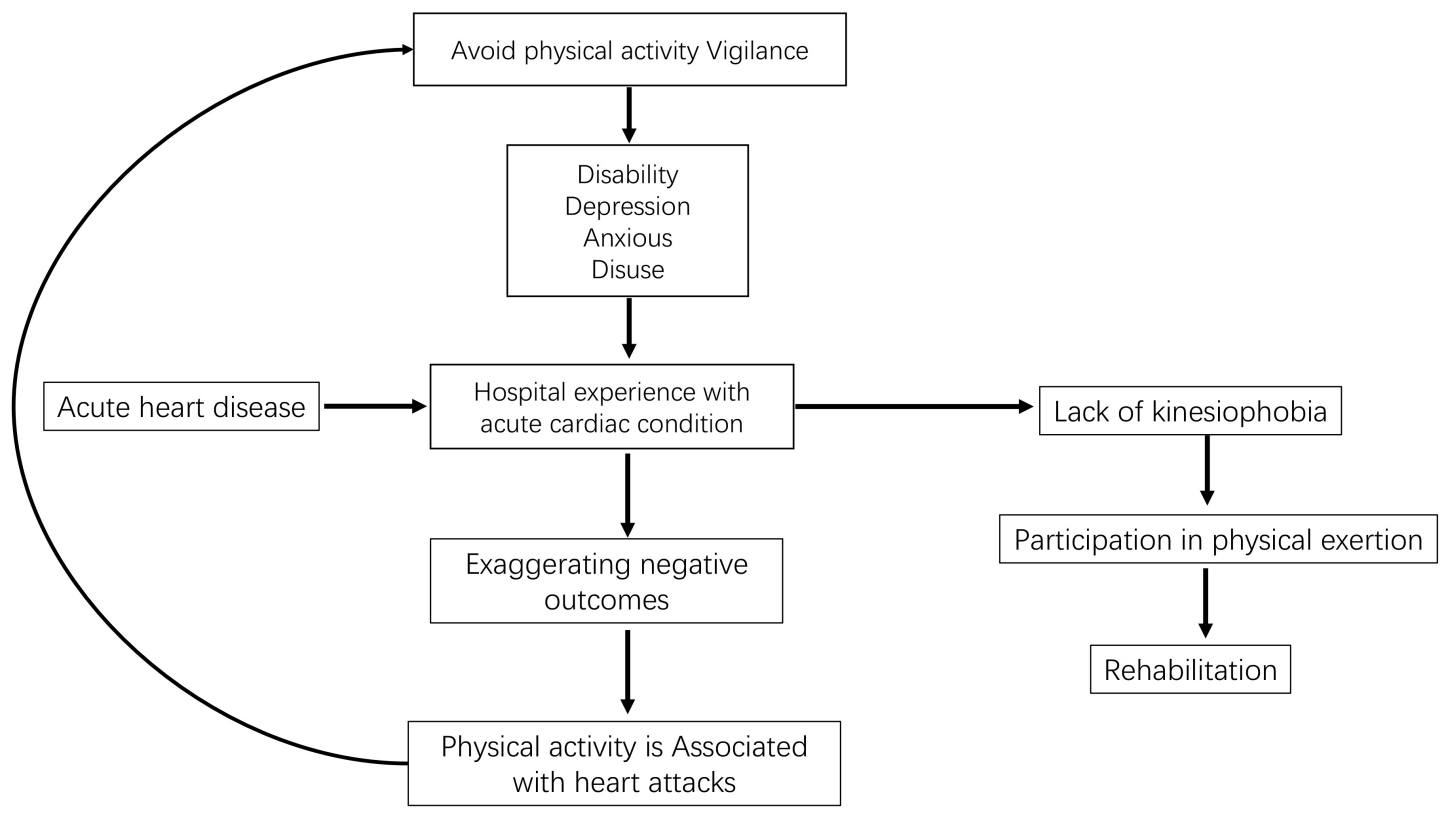
**A possible fear avoidance model in patients with acute heart disease [16]**.

Kinesiophobia was originally proposed by Kori *et al*. [13] and refers to an “excessive, unreasonable and debilitating fear of movement and activity”. Sports phobia has been shown to have an important negative impact on the outcome of rehabilitation, so it is important to study its clinical occurrence [14, 15]. Fear and associated avoidance behavior are typical psychological responses in the early stages after an acute cardiovascular event. Avoidance, as a form of self-protection, can reduce the possibility of adverse reactions caused by stressful events in the short term, while excessive fear of injury may lead to ineffective coping strategies [16]. Based on clinical experience, some AAAD patients may be concerned about the adverse consequences of physical activity after surgery, leading to re-dissection of the aorta, or may be concerned about other complications caused by exercise. Such fears cause patients to be highly vigilant in avoiding exercise that leads, in the long term, to adverse outcomes such as muscle disuse, disability, anxiety and depression. In turn, these physical and psychological symptoms will aggravate the individual’s fear experience of exercise, thus forming a vicious cycle (avoidance strategy), as shown in Fig. 1 (Ref. [16]).

According to the theory of stress process [17], stressors can act on individuals through mediating effects such as family support, coping style and personality characteristics, thus leading to different physiological, psychological and behavioral responses and further affecting the health or disease of individuals. The painful experience caused by symptoms of AAAD is the most common stressor for AAAD patients. Therefore, this study aims to investigate the current situation of exercise fear and related influencing factors of AAAD patients under the guidance of the theory of stress process, so as to provide theoretical reference for improving exercise fear, exercise endurance and disease prognosis of AAAD patients.

## 2. Materials and Methods

### 2.1 Patient Populations

A continuous sampling method was used to identify patients who were first diagnosed with type A aortic dissection in our hospital from January 2019 to December 2021 and successfully implanted with ascending aorta and semi-arch replacement combined with improved three-stent implantation [18]. The surgical details of modified three-branch stenting (MTBSG) have been described in detail before [18], which simply involves the use of modified three-branch stenting to reconstruct the aortic arch, brachiocephalic artery, left common carotid artery, left subclavian artery, and descending aorta. A professional investigator called the patient and informed them of the study. If the patient agreed to participate in the study, a review was scheduled and the relevant questionnaires and tests were completed. Inclusion criteria: ① Age ≥18 years old; ② Patients with stable condition after discharge (3–10 months); ③ Clear consciousness; ④ Informed consent and voluntary participation in research. Exclusion criteria: ① Language communication and body movement disorders (“Physical activity impairment” refers to difficulties or restrictions individuals encounter in performing daily activities, involving issues with movement, coordination, or balance.); ② Patients who could not receive rehabilitation treatment due to severe postoperative complications; ③ Patients were unwilling to continue to cooperate with the study protocol during the study period.

The study was approved by the Ethics Committee of Fujian Medical University Affiliated Union Hospital ([2020] Union Hospital Ethics Review No. 080) and conducted in accordance with the Declaration of Helsinki. We also received written informed consent from subjects or their legal counsels before research commencement.

### 2.2 Data Collection

#### 2.2.1 Sociodemographic and Clinical Data

Sociodemographic and clinical data, including age, sex, marital status, intraoperative conditions and underlying diseases, retrieved from hospital databases. Other data were collected on site, such as education status, living habits, occupational status, regular exercise habits, medical coping style scale, family care index scale, etc. Two trained investigators conducted a questionnaire survey one-on-one. In the questionnaire, the patients made their choices independently, and the researchers accurately recorded each answer. When the patients were unable to fill in the questionnaire by themselves due to factors such as education level, the researchers explained the items for the patients and filled in the questionnaire, and timely remedied the problems such as lack of data filling.

#### 2.2.2 Use of a Pedometer to Assess Muscle Strength

To ensure data collection accuracy, the data collectors were the same members of the research team. On the day of follow-up, one investigator explained the purpose and use of the grip strength test and pedometer to the patients, and two other investigators performed the grip strength test and pedometer correction. Data from grip strength tests were collected during the visit and patients were told to wear pedometers for seven days, after which pedometer data was collected.

### 2.3 Research Tools

#### 2.3.1 General Information Questionnaire

Based on the purpose of the study, through literature research, and expert consultation, a general information questionnaire was prepared for the investigation, including items regarding demographic data, disease history, and hospitalization related medical information.

#### 2.3.2 The Tampa Scale for Kinesiophobia Heart (TSK-SV-Heart)

The TSK-SV-Heart was adapted by Bäck *et al*. [12] from Sweden based on the Exercise fear Scale (Tampa Scale for Kinesiophobia, TSK) for chronic pain patients, and its Cronbach’s α coefficient was 0.78, demonstrating good reliability and validity. The scale contains 17 items and 4 dimensions, namely danger perception, exercise avoidance, exercise fear and functional disorder. Likert 4 scores were used, with 1 = completely disagree, 2 = disagree, 3 = agree and 4 = completely agree respectively. The total score was 17–68, and >37 points could be diagnosed as kinesiophobia. The higher the score was, the more serious the patients’ kinesiophobia.

#### 2.3.3 Muscle Strength Assessment

Grip strength is a good indicator of an individual’s overall muscle strength [19]. In this study, a uniform hand-held grip strength device (Olli 2.26, specification: 15 × 11 × 2.2 cm) was used to measure grip strength twice in each hand, and the higher of the two values was used for data analysis.

#### 2.3.4 Pedometer

Walking is one of the most basic, simple and popular physical activities [20]. In this study, a step-counting App was downloaded on the smartphone of each participant. The accuracy of the pedometer was verified in advance to reduce measurement errors. The average number of steps per day = the sum of steps per week/7.

#### 2.3.5 Numerical Rating Scale (NRS)

When patients were admitted to hospital, they were instructed to use the numerical rating scale (NRS) to assess their pain [21]. The NRS scoring method divides a straight line into 10 segments, with 0 indicating no pain and 10 indicating severe pain. Patients choose a number to represent their degree of pain according to their own feelings, with 1–3 indicating mild pain, 4–6 indicating moderate pain and 7–10 indicating severe pain.

#### 2.3.6 Family Care Index Scale (Adaptation, Partnership, Growth, Affection and Resolve, APGAR)

Designed by Smilkstein [22], its Cronbach’s α coefficient was measured to be 0.80–0.83, with good reliability and validity. The scale includes 5 dimensions, namely fitness, growth, cooperation, affection and affinity density, and the scoring method is Likert 3. “Almost rarely”, “sometimes so” and “often so” were represented by 0–2 respectively. The sum of the dimensions was the total score, which was 10 points. Higher scores indicated a greater degree of family care.

#### 2.3.7 Medical Coping Modes Questionnaire (MCMQ)

The MCMQ, designed by Feifel *et al*. [23], is used to judge the characteristics of coping strategies selected by patients in the face of diseases. There were 20 items in the scale, and the total score was 60, including 3 dimensions, namely, facing, avoidance and yielding. The Likert 4 grading method was adopted. Among the 20 items, items 1, 4, 9, 10, 12, 13, 18 and 19 were scored backwards, and the remaining items were scored forward. The reliability of face, avoidance and yield were 0.69, 0.6 and 0.76, respectively.

#### 2.3.8 General Self-efficacy Scale (GSES)

The GSES was compiled by Schwarzer *et al*. [24] and translated and revised by Wang *et al*. [25]. The Chinese version of the General Self-efficacy Scale is used to measure the self-efficacy of subjects. Cronbach’s α coefficient is 0.87. The scale is a single-dimensional scale consisting of 10 items. The Likert 4 grading method was adopted, with a score of 1 being “very inconsistent”, 2 being “somewhat consistent”, and 3 being “mostly consistent”, and 4 score being “very consistent”. The total score of the scale was calculated by adding the scores of all items and dividing by 10. Higher scores indicated higher levels of self-efficacy.

### 2.4 Data Processing

SPSS 26.0 statistical software (SPSS 26.0: SPSS; Chicago, IL, USA) was used for statistical analysis of the data. The counting data were described using frequency and percentage, and the measurement data were described by mean ± standard deviation. Spearman correlation was used to analyze the correlation between each scale and sports fear score. Inter-group differences in continuous variables were assessed using either the *T*-test or the U-test, while the chi-squared test was employed for categorical variables. After conducting univariate analysis, multivariate analysis was then employed to identify risk factors for kinesiophobia, and *p* < 0.05 indicated statistically significant differences.

## 3. Results 

### 3.1 General Data Analysis

**Fig. 2.**
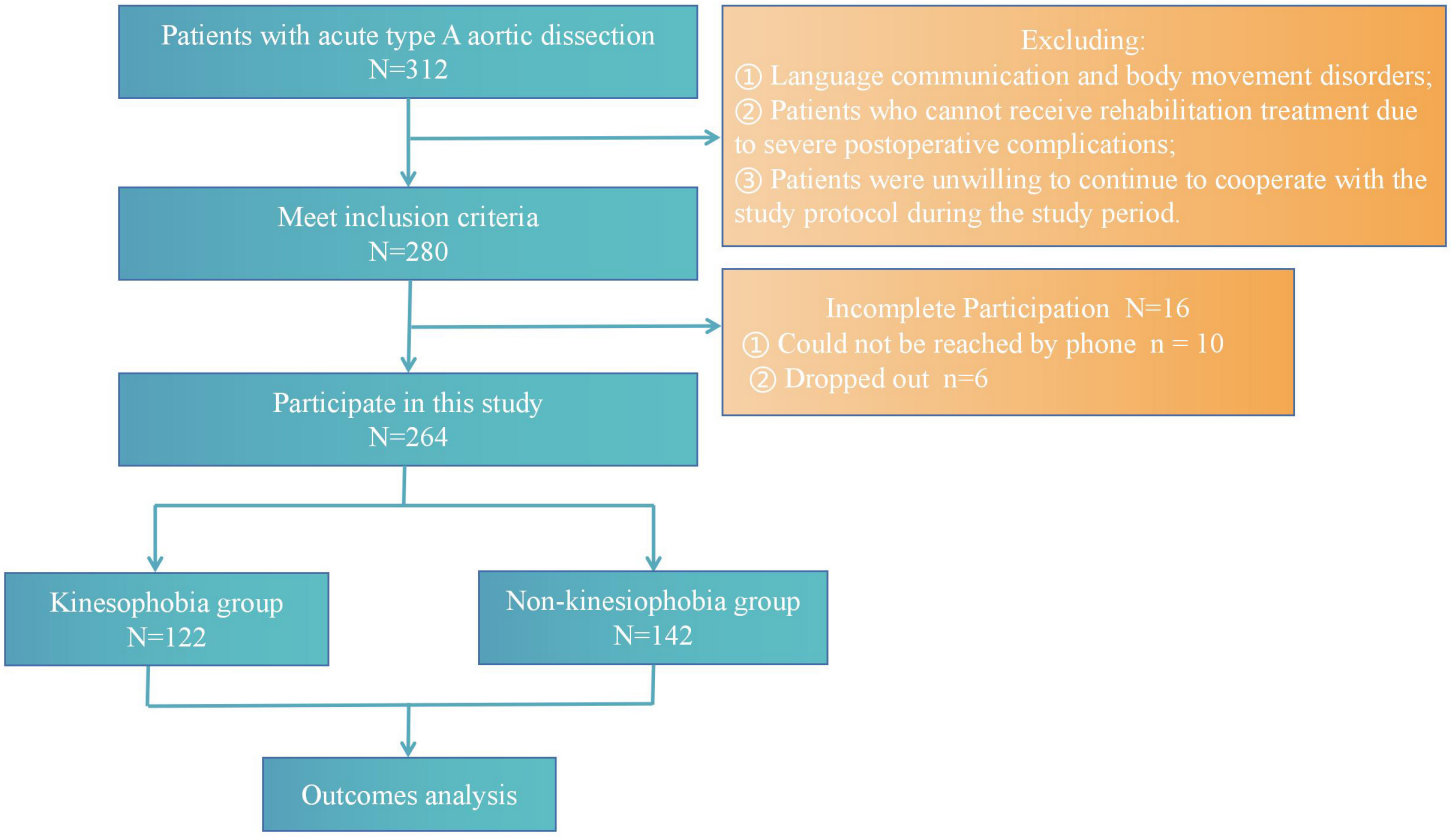
**Flow diagram of the screening and enrollment of study patients**.

**Fig. 3.**
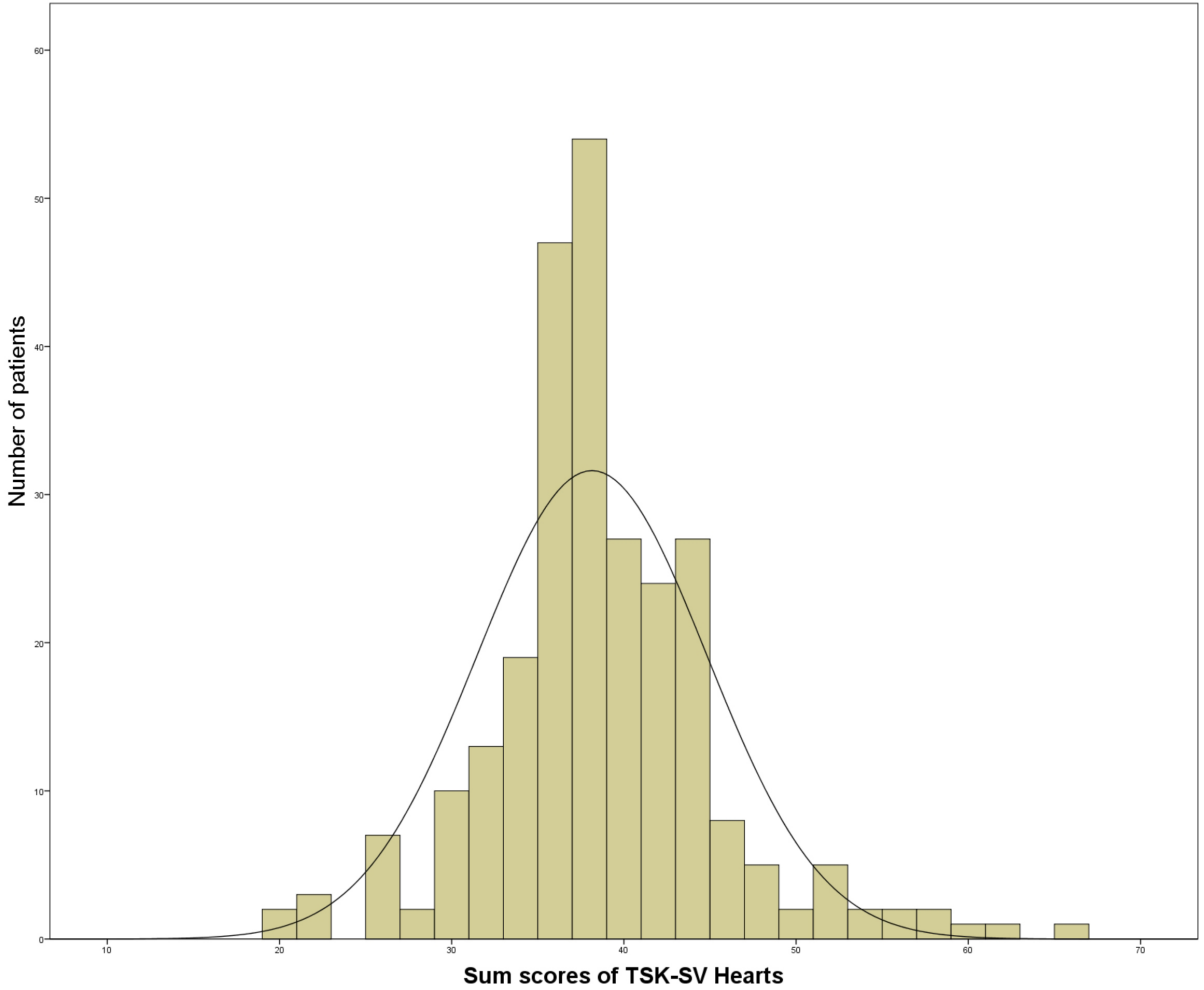
**Frequency distribution of sum scores (17–68 points) of Tampa Scale for Kinesiophobia (TSK-SV) heart in patients with acute type A aortic dissection (AAAD) (N = 264)**.

Among the 312 patients post-AAAD, 280 fulfilled the inclusion criteria (89.74%), 264 questionnaires were filled out validly (84.6%) (Fig. 2), and the average age of patients was 53.33+/–11.85 years. The investigation showed that 122 patients had kinesiophobia, with a prevalence of kinesiophobia post-AAAD of 46.2%, the mean score of postoperative kinesiophobia was 38.15 (6.66) (Fig. 3). Physical activity measurement 6 months after surgery revealed that the pedometer results of patients with kinesiophobia 4275.37 (1067.11)/day were significantly different from those of patients without kinesiophobia 5665.52 (1660.42)/day (*p* < 0.001), and the muscle strength assessment of patients with kinesiophobia were lower, indicating a significant difference between the two groups (*p* < 0.05) (Table 1). All implanted Modified Triple-Branched Stent Graft (MTBSG) were well positioned, and all lateral arm stents were completely unobstructed. No retrograde aortic dissection (AD), neointimal tears at stent edges, or type I intimal leakage were observed. Complete thrombi formation in the pseudolumen was 96.2%.

**Table 1.** **Univariate analysis of patients with and without kinesiophobia**.

Variable		Kinesiophobia (n = 122)	Non-kinesiophobia (n = 142)	*p* value
Age (≥60 y), n (%)		48 (39.3)	32 (22.5)	0.003
Gender (Male), n (%)		95 (77.9)	117 (82.4)	0.36
BMI (kg/m^2^) (≥24), n (%)		69 (56.6)	66 (46.5)	0.10
Marital status (Married), n (%)		112 (93.3)	134 (94.4)	0.41
Education levels (Middle school or under), n (%)		47 (38.5)	87 (61.3)	<0.001
Medical insurance Payment Method (Self-supporting), n (%)		12 (9.8)	10 (7.0)	0.21
Residence (Rural), n (%)		71 (58.2)	75 (52.8)	0.38
Current smoking, n (%)		19 (15.6)	19 (13.4)	0.61
Current drinking, n (%)		29 (23.8)	40 (28.2)	0.42
Pain, n (%)		107 (87.7)	125 (88.0)	0.94
NRS score (SD)		7.76 (3.13)	7.98 (3.18)	0.58
Steps/day (pedometer) (SD)		4275.37 (1067.1)	5665.52 (1660.4)	<0.001
Grip strength test				
	Left hand grip strength test (kg) (SD)	29.80 (6.6)	32.54 (7.3)	0.002
	Right hand grip strength test (kg) (SD)	31.84 (8.6)	35.46 (8.6)	0.001
Occupational status				
Preoperative physical labor		51 (51.5)	48 (48.5)	0.566
Postoperative physical labor		33 (42.9)	44 (57.1)	0.483
Exercise habits				
	Regular exercise before surgery	22 (50.0)	22 (50.0)	0.581
	Regular exercise after surgery	28 (53.8)	24 (46.2)	0.218
Diabetes mellitus, n (%)		6 (4.9)	10 (7.0)	0.47
Hypertension, n (%)		84 (68.9)	94 (66.2)	0.65
Hyperlipidemia, n (%)		17 (13.9)	10 (7.0)	0.07
Marfan syndrome, n (%)		2 (1.64)	5 (3.5)	0.34
Heart failure, n (%)		3 (1.1)	2 (1.4)	0.53
Hemoglobin (g/L) (SD)		127.10 (13.8)	125.46 (15.4)	0.37
EF (%) (SD)		61.9 (6.1)	61.99 (7.5)	0.89
MCMQ				
	Facing	19.2 (2.5)	22.4 (3.3)	<0.001
	Avoidance	19.5 (2.4)	16.3 (2.7)	<0.001
	Yielding	11.9 (2.2)	9.5 (1.8)	<0.001
APGAR		6.4 (1.1)	7.9 (1.3)	<0.001
GSES		22.1 (3.1)	24.2 (4.2)	<0.001

BMI, body mass index; NRS, numerical rating scale; EF, left ventricular ejection fraction; MCMQ, Medical Coping Modes Questionnaire; APGAR, adaptation, partnership, growth, affection and resolve, family care index scale; GSES, general self-efficacy scale; SD, standard deviation. Continuous normally distributed variables were expressed as mean (standard deviation) and categorical data are given as the counts and percentage (n, %).

Although most patients still had residual abdominal aortic dissection, no significant abdominal aortic enlargement was observed compared to preoperative findings. The prevalence of cerebral complications was low due to the short time of circulatory stop and low-flow cerebral perfusion, with 10 cases of temporary neurological dysfunction (3.8%). Other basic characteristics and surgical data are shown in Tables 1,2. There was no significant difference in medical variables between the two groups (*p* > 0.05), indicating that the baseline risk levels and surgical data of patients in both groups were consistent (Tables 1,2).

**Table 2.** **Surgical data and Last Follow-Up data on patients with and without kinesiophobia**.

Valuables		Kinesiophobia (n = 122)	Non-kinesiophobia (n = 142)	*p* value
Surgical data				
	ICU treatment days (SD)	3.6 (1.34)	3.88 (1.64)	0.14
Intraoperative time				
	Operative time (min) (SD)	304.5 (56.8)	308.2 (51.9)	0.58
	Cardiopulmonary bypass time (min) (SD)	148.4 (31.6)	146.5 (37.1)	0.66
	Aortic clamp time (min) (SD)	55.1 (16.7)	54.3 (15.9)	0.69
	Low flow cerebral perfusion time (min) (SD)	16.7 (4.3)	16.4 (5.9)	0.64
Concomitant arotic root procedure				
	Bentall (%)	22 (18.0)	29 (20.4)	0.62
	Aortic valve replacement (%)	2 (1.6)	3 (2.1)	0.86
	Coronary artery bypass surgery (%)	9 (7.4)	11 (7.7)	0.91
	David (%)	3 (2.5)	4 (2.8)	0.83
	Valsalva sinus forming (%)	25 (20.5)	28 (19.7)	0.88
	Temporary neurologic dysfunction (%)	3 (2.5)	7 (4.9)	0.30
Last Follow-Up data				
	EF (%) (SD)	62.01 (7.75)	63.23 (6.20)	0.16
	Aortic insufficiency (yes), n (%)	1 (0.8)	3 (2.1)	0.39
	False lumen thrombus (yes), n (%)	116 (95.1)	138 (97.2)	0.37

EF, left ventricular ejection fraction; SD, standard deviation; ICU, intensive care unit. Continuous normally distributed variables were expressed as mean (standard deviation) and categorical data are given as the counts and percentage (n, %).

### 3.2 Univariate Analysis of Postoperative Kinesiophobia in Patients with AAAD

Descriptive results and the differences between high versus low levels of kinesiophobia for each variable are presented in Tables 1,2.

### 3.3 Family Care Index, Medical Coping Style and General Self-Efficacy Score

In order to facilitate the interpretation of the results in Table 1, the relationships between kinesiophobia and AAAD variables were reported as correlations. The Spearman correlation analysis results showed that there is a correlation between postoperative APGAR (Adaptation, Partnership, Growth, Affection and Resolve), MCMQ (Medical Coping Modes Questionnaire), GSES (General Self-efficacy Scale), and kinesiophobia levels in AAAD patients. (*p* < 0.01). The total family care index score of the study participants was 7.2 (1.43), indicating good family function. The scores of medical coping styles were as follows: facing 20.92 (3.36) > avoidance 17.76 (3.01) > submission 10.60 (2.30), and the total score of postoperative general self-efficacy was 23.2 (4.0), presenting a moderate level. There were significant differences between the two groups regarding family care, medical coping style and self-efficacy (*p* < 0.001) (Table 3).

**Table 3.** **The scores of family care index, medical coping style and general self-efficacy scale and their correlation with kinesiophobia**.

Variable		Score	R value	*p* value
MCMQ				
	Facing	20.92 (3.36)	–0.43	<0.001
	Avoidance	17.76 (3.01)	0.51	<0.001
	Yielding	10.60 (2.30)	0.50	<0.001
APGAR		7.2 (1.43)	–0.42	<0.001
GSES		23.23 (3.85)	–0.25	<0.001

MCMQ, Medical Coping Modes Questionnaire; APGAR, adaptation, partnership, growth, affection and resolve, family care index scale; GSES, general self-efficacy scale. Continuous normally distributed variables were expressed as mean (standard deviation).

### 3.4 Results of Multiple Logistic Regression Analysis

Considering the existence of fear of motility as a dependent variable, the related variables conforming to *p* < 0.1 in the univariate analysis results were included in the multivariate logistic regression analysis (Table 4), and 11 variables finally met the inclusion criteria: general demographic data: Age (≥60 years old), hyperlipidemia (yes), education level (middle school or under), steps/day, left hand grip strength test, right hand grip strength test, scores of family care index, scores of various dimensions of medical coping style, total scores of general self-efficacy. Continuous variables were input with original values, and classified variables were assigned values. Multivariate logistic regression analysis showed education level (odds ratio, OR = 3.97, 95% CI: 1.27–12.39, *p* < 0.05), general self-efficacy (OR = 0.74, 95% CI: 0.63–0.88, *p* < 0.05), family care index (OR = 0.27, 95% CI: 0.15–0.47, *p* < 0.001), facing style (OR = 0.69, 95% CI: 0.56–0.85, *p* < 0.001). Two variables increased the level of kinesiophobia: avoidance style (OR = 1.65, 95% CI: 1.32–2.05, *p* < 0.001) and yielding style (OR = 2.10, 95% CI: 1.58–2.80, *p* < 0.001) were risk factors for kinesiophobia in patients with acute type A aortic dissection (*p* < 0.001).

**Table 4.** **Multivariate logistic regression analysis of kinesiophobia in AAAD patients**.

Independent variables		B	S.E.	Wald	*p*	Exp (B)	Lower 95% CI for Exp (B)	Upper 95% CI for Exp (B)
Age (≥60 y)		0.48	0.64	0.55	0.46	1.61	0.46	5.7
Hyperlipidemia		0.20	2.10	0.01	0.92	1.22	0.02	69.3
Education levels (Middle school or under)		1.38	0.58	5.62	0.02	3.97	1.27	12.39
	Left hand grip strength test (kg)	0.01	0.03	0.22	0.64	1.01	0.96	1.07
	Right hand grip strength test (kg)	–0.02	0.03	0.43	0.51	0.98	0.93	1.03
Steps/day (pedometer)		–0.00	0.00	16.51	<0.001	0.10	0.10	1.0
APGAR		–1.30	0.29	20.03	<0.001	0.27	0.16	0.48
Facing		–0.37	0.11	12.66	<0.001	0.70	0.56	0.85
Avoidance		0.50	0.11	19.75	<0.001	1.65	1.32	2.05
Yielding		0.74	0.15	26.42	<0.001	2.10	1.58	2.80
GSES		–0.30	0.09	11.44	0.001	0.74	0.63	0.88

Age: <60 = 1, ≥60 = 2; High blood lipid: yes = 1, no = 0; Education: Junior high school or below = 1, senior high school or above = 2; Family care index, medical coping style questionnaire and general self-efficacy were input with original values. APGAR, Adaptation, Partnership, Growth, Affection and Resolve, Family care index scale; GSES, General Self-efficacy Scale; S.E., standard error; Exp, exponentiation; B, regression coefficient.

## 4. Discussion

The cross-sectional results of this study found that 46.2% of patients who survived AAAD exhibited varying degrees of kinesiophobia after successful surgery, with high levels of patients with kinesiophobia with lower education levels, negative coping styles, lower family care indices and generally lower self-efficacy were associated with postoperative kinesiophobia.

In our study, 46.2% of patients with AAAD suffered from kinesiophobia, which was higher than the results of Bäck *et al*. [12] on the prevalence of kinesiophobia in 332 patients with coronary heart disease (20%). The disparate results may be explained by the different nature of the disease. In the present study, 87.9% of the patients had severe pain at the time of disease onset, which easily resulted in traumatic fear memories. Many patients were unable to cope with their fear, leading to long-term avoidance of physical activities and exercise. According to relevant literature, even if surgical intervention is performed at an early stage, the mortality rate of recurrence and the prevalence of surgical complications are high [26]. Some patients avoid physical activity and exercise for fear of disease recurrence caused by activities. The specific psychological process of fear of exercise in patients with AAAD could be explored by qualitative studies in the future.

Patients with a fear of movement exhibited lower postoperative muscle strength and physical activity compared with patients without kinesiophobia (*p* < 0.01) in a previous study of neck trauma muscle activation which also found that the muscle performance negatively correlated with kinesiophobia [27]. This study found that patients with lower education levels had lower levels of kinesiophobia, which is consistent with the results of Luo X [28], but differs from some previous studies, possibly due to differences in diseases and study populations. The results of Alsaleem *et al*. [29] indicated that kinesiophobia was not related to education level, while the results of Tan *et al*. [30]. showed that patients with lower education levels had higher levels of kinesiophobia. There may be two reasons for this difference. First, patients with lower education levels may have a lower level of disease awareness, insufficient understanding of the risks and consequences of the disease, and are more likely to accept simple and direct explanations and treatment plans, thereby experiencing less fear of the disease. Second, patients with higher education levels may have a deeper understanding of the risks and consequences of the disease, leading to overinterpretation and exaggeration of the risks of surgery [28], thus causing more worry. Further research could consider using more refined methods of classifying education levels and combining them with cultural cognitive factors for analysis to better understand the relationship between education level and kinesiophobia.

Our results showed a significant difference between the two groups in terms of self-reported activity (number of steps), *p* < 0.001, suggesting the possibility that AAAD patients avoid physical activity. According to reports, an average of 10,000 steps per day appears to be a reasonable estimate for the daily activity of apparently healthy adults, with less than 5000 steps per day serving as an index for a sedentary lifestyle [31]. In our study, patients with kinesiophobia had an average postoperative step count of 4275.37 (1067.1), significantly lower than this level. This may suggest that they are substituting activities that provoke fear with less intimidating ones, such as sedentary behaviors. However, Maria Bäck *et al*. [12] showed in their study on kinesiophobia in patients with coronary heart disease that there was no significant difference between patients with kinesiophobia and those without kinesiophobia in terms of low-intensity activity and self-reported activity in activity diaries. The reasons for this difference may be related to factors such as disease characteristics or the degree of participation in postoperative cardiac rehabilitation. The effect of physical activity level on kinesiophobia must be confirmed in future studies.

Our study showed that general postoperative self-efficacy was negatively correlated with kinesiophobia in AAAD patients (Table 3). The results of multifactor logistic regression logic analysis showed that general self-efficacy was the influencing factor for the occurrence of kinesiophobia in postoperative AAAD patients. People with higher self-efficacy were more likely to start and continue activities conducive to recovery, while the opposite was true for people with lower self-efficacy [32]. Studies have shown that self-efficacy is positively correlated with long-term adherence to physical activity during post-discharge CR [33]. Another study [25] showed that self-efficacy is an important risk factor for sports phobia after total knee replacement [OR = 1.4]. Zelle *et al*. [15] also believe that self-efficacy is closely related to sports phobia and may be an important factor in the relationship between sports phobia and physical activity. Therefore, this study found that the influence of exercise fear on physical activity can be explained by a low sense of self-efficacy to a large extent [34]. Positive and healthy self-efficacy beliefs can make patients more confident to regulate emotions and induce compliance with brain movements. Meanwhile, in this study, 81.1% of the subjects were in junior high school or below, and their ability to accept, understand and actively acquire disease knowledge was low. The more adverse consequences they had of their illness, the lower their levels of self-efficacy. Second, self-efficacy can influence physical activity behaviors through the development and use of self-regulating behaviors. Patients with low self-efficacy have more difficulty facing painful traumatic memories and are more likely to adopt negative coping strategies to cope with the stress caused by kinesiophobia, leading to further fear of physical activity. Given this, it is necessary for healthcare providers to consider self-efficacy as a contributing factor to exercise phobia and establish self-efficacy enhancement strategies to increase the chances of physical activity in postoperative AAAD patients.

We also found that negative coping styles are a significant risk factor for dyskinesia. The stress coping model proposed by Feifel *et al*. [23] shows that coping styles play an important role in disease outcomes in addition to patients and disease characteristics. This result is consistent with the results of Worm [35], who found that negative coping was associated with kinesiophobia and increased rates of long-term disability and depression. Somers *et al*. [36] also concluded that individuals who experience pain-related fears are likely to engage in avoidance behaviors, especially avoiding sports and physical activities. Although these patients had successful surgery and improved function after surgery, they still struggled to erase traumatic memories, leading to anxiety and other difficult mood changes. In addition, patients are concerned about the risk of elevated blood pressure due to exercise during CR because they are uncertain about their future health and safe level of exercise after surgery. Thus, more negative coping strategies can be used to deal with the stress caused by sports phobia, leading to higher pain intensity and further fear of physical activity.

In addition to the above risk factors, we found that less family support was associated with an increased prevalence of kinesiophobia and that APGAR was significantly negatively associated with kinesiophobia scores. Multivariate analysis suggested that APGAR was a protective factor for postoperative kinesiophobia in AAAD patients. Birtwistle *et al*. [37] also demonstrated that family support is closely related to physical activity (PA) behavior. Earlier studies have found that family support is associated with greater anxiety, depression and reduced mobility in people with CABG (Coronary Artery Bypass Grafting). Family members can communicate CR information to patients, encourage positive behaviors, and provide partner-based forms of PA to facilitate PA participation [37]. High levels of family support are associated with higher CR attendance and even longer survival rates; More family support predicted positive changes in mental health, while low levels of family support were associated with poorer perceptual status and physical dysfunction [38, 39]. Early studies have confirmed that integrating the family into CR helps promote physical actively-related interactions [37] and reduces the occurrence of sports kinesiophobia. Therefore, there is strong evidence that family support is a key factor in PA and can reduce the prevalence of sports kinesiophobia. However, there are some challenges within the family, including the family’s “over-involvement” and the family’s own health beliefs, which can negatively affect patients, and the role of family caregivers in AAAD patients requires further efforts.

The main strength of this study is that it produced cross-sectional findings showing the influence of related factors on kinesiophobia in AAAD patients. The study found that exercise phobia was present in 46.2% of AAAD patients. In addition, the impact on recovery outcomes in AAAD patients was determined by clinical variables that represent components of the theory of processes of stress action. In light of these findings, kinesiophobia needs to be considered in the rehabilitation of AAAD patients and given priority in future studies.

## 5. Limitations

The following limitations were also considered in this study. Firstly, this was a single-center study with a limited sample size, which may affect the statistical results. Future research directions should involve larger sample sizes and employ a more rigorous multi-center design to further validate our conclusions. Secondly, recall bias may occur in remote telephone collection of questionnaire information. More rigorous data collection methods should be developed for subsequent studies, such as face-to-face interviews or real-time online surveys. Thirdly, our study only examined some factors that may affect kinesiophobia, but did not take into account the influence of preoperative factors, particularly anxiety and sleep state, which have been proven in other studies to increase patients’ perception of pain or the occurrence of kinesiophobia [40, 41]. In summary, we advocate for future studies to build upon these identified limitations, utilizing more comprehensive and diverse research approaches to strengthen the reliability and applicability of our conclusions.

## 6. Conclusions 

Patients with AAAD exhibit a high prevalence of postoperative kinesiophobia. Patients’ education level, medical coping style, family care index and general self-efficacy are related to the occurrence of postoperative kinesiophobia. Therefore, the existence of kinesiophobia should be considered in the CR of patients with AAAD.

## Data Availability

The data that support the findings of this study are available from Fujian Cardiac Medical Center but restrictions apply to the availability of these data, which were used under license for the current study, and so are not publicly available. Data are however available from the authors upon reasonable request and with permission of Fujian Cardiac Medical Center.

## Abbreviations

AAAD, Acute type A aortic dissection; AAD, Acute aortic dissection; CR, Cardiac rehabilitation; MTBSG, Modified Triple-Branched Stent Graft; AD, Aortic dissection; TSK-SV-Heart, The Tampa Scale for kinesiophobia Heart; PA, Physical activity; NRS, Numerical Rating Scale; GSES, General Self-efficacy Scale; MCMQ, Medical Coping Modes Questionnaire; APGAR, Adaptation, Partnership, Growth, Affection and Resolve, Family care index scale.
